# The multiple sclerosis exposome: environmental determinants, immunological convergence, and the Gulf Region as a natural test case

**DOI:** 10.3389/fimmu.2026.1941832

**Published:** 2026-10-05

**Authors:** Eslam Shosha, Najeeb Qadi

**Affiliations:** 1 Neuroscience Center of Excellence, King Faisal Specialist Hospital and Research Center, Riyadh, Saudi Arabia; 2 Departments of Medicine, Neurology Division, McMaster University, Hamilton, ON, Canada; 3 College of Medicine, Alfaisal University, Riyadh, Saudi Arabia

**Keywords:** air pollution, environmental risk factors, exposome, gene–environment interaction, gulf cooperation council, heavy metals, multiple sclerosis, organic solvents

## Abstract

**Background:**

The multiple sclerosis (MS) exposome is widely invoked but rarely measured; grouping environmental exposures together obscures large differences in evidence quality. Much of the existing gene–environment evidence originates from genetically and environmentally homogeneous northern European cohorts, leaving open whether it generalizes elsewhere. The Gulf Cooperation Council (GCC) region is a largely untested candidate for such a test: ambient particulate concentrations there routinely exceed international guideline values, the dominant particulate sources and composition differ from those characterized in European studies, and the region’s HLA-DRB1 allele distribution diverges substantially from the populations in which most gene–environment interaction evidence has been generated.

**Approach:**

This narrative review appraises exposure domains against six pre-specified criteria—design, exposure assessment, temporality, dose–response, confounding control, and gene–environment interaction—and reports effect estimates. Search strategy, selection criteria, and appraisal limitations are stated explicitly.

**Key findings:**

Inhalational exposures carry the most coherent signal. Pooled long-term particulate estimates are modest but consistent (PM_2.5_ HR 1.21, 95% CI 1.07–1.38); combustion-related NO*_x_* shows dose dependence and interaction with HLA-DRB1*15:01; organic solvents and industrial dust show similar HLA-restricted interaction patterns. Two discordances temper this: the two largest prospective cohorts were null, and Mendelian randomization does not support a causal effect of smoking. Much of the interaction evidence derives from related Swedish datasets and is not independently replicated. Heavy metals rest on cross-sectional biomonitoring vulnerable to reverse causation; persistent organic pollutants contribute a mixture-level signal. Contemporary evidence does not support a Gulf-War-attributable increase in MS.

**Working model:**

Chemically diverse exposures converge toward a limited set of immunological nodes—aryl hydrocarbon receptor activation, short-chain fatty acid depletion, EBNA1/GlialCAM mimicry, and oxidative impairment of regulatory T cells—each conditioned by HLA-restricted susceptibility.

**Implications:**

We set out why the Gulf Cooperation Council region may be an informative setting for testing this model, which designs and immunological endpoints would test it, and what would falsify it.

## 1 Introduction

### 1.1 The problem

Multiple sclerosis (MS) is an inflammatory, demyelinating, and neurodegenerative disease of the central nervous system and a leading cause of non-traumatic neurological disability in young adults. The third edition of the Atlas of MS estimated 2.8 million people living with MS worldwide in 2020, rising in every world region since 2013 (1); the 2023 core data update raised this to approximately 2.9 million (2). This shift has occurred over decades rather than generations. It, therefore, cannot be attributed to changes in population genetics and instead implicates environmental and lifestyle factors acting on a susceptible background, most prominently HLA-DRB1*15:01 (3).

Four determinants are well supported. Epstein–Barr virus (EBV) infection now approaches the status of a necessary cause: in more than 10 million US military personnel, seroconversion carried a hazard ratio of 32.4 (95% CI 4.3–245.3) for subsequent MS, with a median lag of roughly 7 years, no comparable signal for other viruses, and a rise in serum neurofilament light chain only after seroconversion (4, 5). Reduced ultraviolet exposure and low vitamin D status track susceptibility, particularly in early life (6, 7). Smoking raises the risk of onset and worsens course (8, 9). Adolescent obesity has emerged as a fourth contributor, reinforced by recent comprehensive syntheses (10, 11). Mendelian randomization has been used to test all four; as Section 3 sets out, it supports causality for vitamin D and body mass index but not, on current instruments, for smoking (12–25).

Beyond these, a broader set of exposures has attracted interest: ambient air pollution and combustion gases, occupational dust and organic solvents, heavy metals, persistent organic pollutants, diet and the gut microbiome, residential proximity to industry, and large-scale environmental disruption associated with armed conflict. Evidence quality differs sharply. Some rests on large populations with objective exposure modeling. Other evidence rests on cross-sectional biomonitoring, retrospective exposure histories, or ecological comparison.

Contemporary environmental epidemiology situates such factors within the exposome: the cumulative burden of environmental influences across the life course and their interaction with inherited susceptibility, as originally formulated by Wild and subsequently extended to internal as well as external exposure (26–28). **Figure 1** summarizes this life-course framework as applied to MS. The framework’s value is that it takes correlated exposure seriously. Its risk is that it can license the promotion of weak associations by association with strong ones.

**Figure 1:**
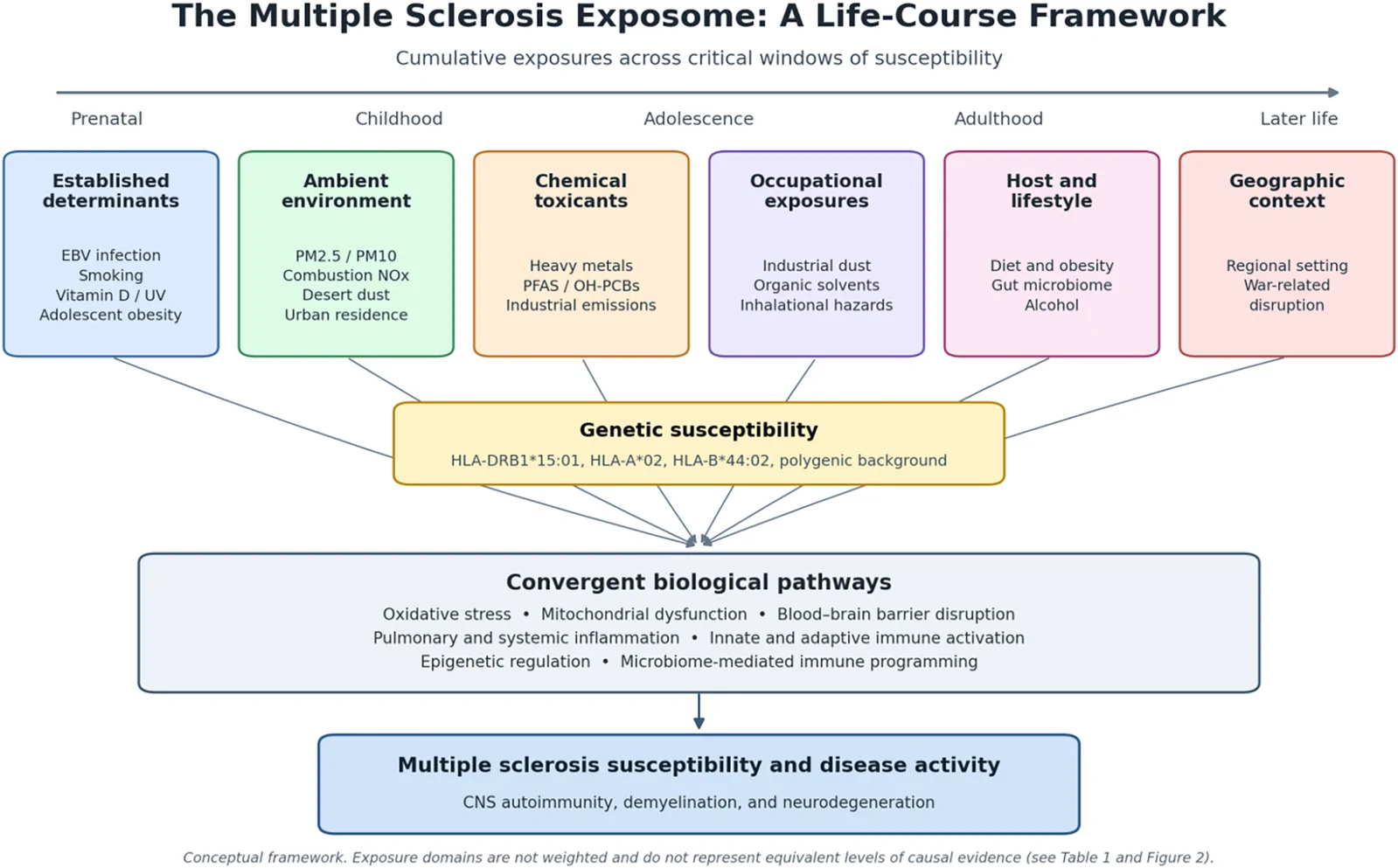
The multiple sclerosis exposome: a life-course framework. Established and emerging exposure domains may act during critical windows of susceptibility and interact with genetic background, including HLA-DRB1*15:01, HLA-A*02, and HLA-B*44:02. Diverse exposures converge toward shared biological pathways, including oxidative stress, mitochondrial dysfunction, blood–brain barrier disruption, pulmonary and systemic immune activation, epigenetic regulation, and microbiome-mediated immune programming. The framework is conceptual: domains are not weighted, and inclusion does not imply equivalent causal evidence. Comparative evidence strength is given in **Table 1** and effect estimates in **Figure 2**.

Much of this evidence base has been generated in northern European and North American cohorts, in which HLA-DRB1*15:01 is common; gene–environment interaction has, therefore, been characterized against a relatively homogeneous genetic background, and ambient particulate exposure rarely approaches the levels reached elsewhere. The Gulf Cooperation Council (GCC) region offers a marked contrast on each of these axes and is used throughout this review as a worked illustration of how the appraisal framework in Section 2.4 applies to a non-European setting. Ambient particulate concentrations in Gulf cities routinely exceed World Health Organization guideline values, driven by a mixture of crustal desert dust and an anthropogenically enriched, combustion-derived fraction that is compositionally distinct from the particulate matter characterized in most European exposure–response studies. MS in the region was historically classified as low risk under early prevalence schemas; national registries in Saudi Arabia and Kuwait now document rising prevalence and incidence, and the region’s HLA-DRB1 allele distribution and haplotype structure differ substantially from those of northern European populations. Long-term ambient air quality monitoring, speciated particulate composition data, and functioning national MS registries already exist for parts of the region, although they have not yet been linked to one another or to genotyping. This combination of an extreme and compositionally distinct exposure, a divergent genetic background, rising disease occurrence, and partial existing infrastructure is what motivates the regional test case developed in Section 10, and it is introduced here because it shapes how the evidence reviewed in the sections that follow should be read.

### 1.2 A working model, stated so that it can be tested

Reviews of this literature typically enumerate exposures. Enumeration is not a model, and it cannot be refuted. We, therefore, state a single working model at the outset and organize the review around it. The model makes three linked claims.

Claim 1 (exposure): Among emerging exposures, the inhalational route carries the most internally coherent evidence because it is the route for which objective exposure assessment, dose–response, and recurrent gene–environment interaction signals have all been demonstrated, although much of the interaction evidence comes from related Swedish datasets (Sections 4 and 5).

Claim 2 (mechanism): Chemically diverse exposures do not require chemically diverse mechanisms. A small number of molecular nodes—aryl hydrocarbon receptor (AhR) ligation, short-chain fatty acid (SCFA) availability, EBV-associated antigenic mimicry, and redox-dependent regulatory T-cell (Treg) function—can plausibly translate several unrelated agents into the same effector–regulatory shift, conditioned by HLA genotype (Section 8).

Claim 3 (test): If Claims 1 and 2 hold, then a population with a far higher inhalational exposure ceiling, a different particulate composition, and a different HLA background should show a detectable dose–response and a genotype-restricted interaction. If it does not, the model is weakened. The GCC region offers these contrasts and already has much of the infrastructure needed to test them (Section 10).

The order of the review follows this logic: evidence (Sections 3–7), mechanism (Section 8), measurement (Section 9), and then the proposed test with its falsifying conditions (Section 10).

### 1.3 Scope and what this review deliberately excludes

The MS exposome is large enough that any single review must choose. We restrict attention to non-infectious environmental and lifestyle exposures for which quantitative human effect estimates exist, plus EBV, which is included only as the evidential benchmark against which emerging exposures are judged. Four bodies of work are deliberately outside the scope and are not reviewed here: infectious agents other than EBV; psychosocial stress, socioeconomic position, and adverse life events as exposures in their own right (they appear only as confounders); ionizing and non-ionizing radiation; and the large literature on exposures as modifiers of treatment response in established disease. Section 7 covers diet and the microbiome only insofar as they bear on the working model; the dietary literature, as a whole, is not surveyed. These exclusions are stated so that the reader can judge what the review does not cover, rather than inferring omission from silence.

Throughout, evidence relating to MS susceptibility and disease onset is distinguished from evidence relating to disease activity, relapse, or progression. These outcomes may involve different exposure windows and biological mechanisms. An exposure that influences initial autoimmune susceptibility need not affect established disease activity, and the converse also holds. Where the distinction is uncertain in a primary study, this is noted.

## 2 Methods

### 2.1 Review type and justification

This is a narrative review, prepared with reference to SANRA guidance for narrative review quality (29). A systematic review was not undertaken, for a reason specific to this question rather than for convenience. The review’s purpose is to compare evidential strength *across* heterogeneous exposure domains—ambient pollution, occupational agents, metals, persistent pollutants, diet, and conflict-related exposure—whose primary studies use incommensurable designs, exposure metrics, and effect measures. A single systematic protocol capable of screening all of these domains would either be so broad that its yield could not be appraised consistently or so narrow that it would answer a question about one exposure rather than about the exposome. Quantitative pooling across these domains would be misleading for the same reason, as the heterogeneity within a single domain already demonstrates (*I*² = 95% for PM_2.5_) (30). What this design cannot deliver is reproducible selection, and that limitation is stated in Section 2.4 rather than minimized.

### 2.2 Search strategy

Literature was identified through searches of PubMed/MEDLINE, Scopus, and Google Scholar from database inception to July 15, 2026, without language restriction. The copy-pasteable core PubMed strategy was:

(“multiple sclerosis”[MeSH Terms] OR “multiple sclerosis”[tiab] OR “demyelinating disease*”[tiab]) AND (“exposome”[tiab] OR “environmental exposure*”[tiab] OR “air pollution”[MeSH Terms] OR “particulate matter”[tiab] OR “PM_2.5_”[tiab] OR “PM_10_”[tiab] OR “nitrogen dioxide”[tiab] OR “nitrogen oxides”[tiab] OR “carbon monoxide”[tiab] OR “organic solvent*”[tiab] OR “occupational exposure”[MeSH Terms] OR “occupational dust”[tiab] OR “industrial exposure*”[tiab] OR “oil refiner*”[tiab] OR “petrochemical*”[tiab] OR “heavy metal*”[tiab] OR “lead”[tiab] OR “mercury”[tiab] OR “cadmium”[tiab] OR “arsenic”[tiab] OR “PFAS”[tiab] OR “perfluoroalkyl”[tiab] OR “polychlorinated biphenyl*”[tiab] OR “persistent organic pollutant*”[tiab] OR “gut microbiome”[tiab] OR “microbiota”[tiab] OR “diet”[tiab] OR “short-chain fatty acid*”[tiab] OR “obesity”[tiab] OR “vitamin D”[tiab] OR “smoking”[tiab] OR “Epstein-Barr”[tiab] OR “Mendelian randomization”[tiab] OR “gene-environment interaction”[tiab] OR “war”[tiab] OR “Gulf War”[tiab])

For Section 10 and the regional background, the following geographic block was appended to the core strategy:

(“Saudi Arabia”[tiab] OR “Gulf Cooperation Council”[tiab] OR “Kuwait”[tiab] OR “Qatar”[tiab] OR “United Arab Emirates”[tiab] OR “Bahrain”[tiab] OR “Oman”[tiab] OR “Middle East”[tiab])

Equivalent term combinations were adapted for Scopus (TITLE-ABS-KEY) and Google Scholar; because Boolean capability is more limited in Google Scholar, it was used chiefly for citation chasing. Reference lists of retrieved reviews, meta-analyses, and major epidemiological studies were also hand-searched. Exact historical execution logs were not retained prospectively, and no retrospective record counts or platform-specific search logs are presented as though they had been recorded at the time.

### 2.3 Selection of sources

Records were eligible if they reported original human data on an environmental or lifestyle exposure and an MS outcome (incidence, prevalence, susceptibility, relapse, MRI activity, or progression) or if they were a systematic review, meta-analysis, or Mendelian randomization study of such data. Mechanistic studies in animals or human cells were eligible only for Section 8 and only where they addressed a molecular node already implicated by human epidemiological evidence. We excluded case reports, conference abstracts without a corresponding full paper, and studies without an extractable effect estimate or a quantified exposure contrast, except where a study is cited to illustrate a design limitation.

Within each domain, priority was given, in this order, to (i) systematic reviews and meta-analyses, (ii) Mendelian randomization studies, (iii) large population-based cohort or registry studies, and (iv) primary studies with objective exposure assessment. Where several studies addressed the same exposure with the same design, the largest and most recent was cited, with earlier work cited where it established the finding or where it disagrees. This is a citation rule, not a screening rule: it determines which of the eligible studies are discussed, and it is stated here so that the reader can see why a given domain is represented by a small number of references.

Language: no language restriction was applied at the search stage. All studies that met the eligibility criteria and were finally cited are published in English. We report this rather than implying broader coverage than was achieved; relevant non-English literature, particularly from the Middle East and North Africa region, may, therefore, be under-represented, and this is a genuine limitation of the search rather than a property of the field.

### 2.4 Appraisal framework and what it is not

Evidence was appraised against six criteria applied consistently across domains: (i) study design and its vulnerability to reverse causation, (ii) objectivity of exposure assessment, (iii) temporality of exposure relative to disease onset, (iv) evidence of dose–response, (v) adequacy of adjustment for smoking, socioeconomic status, urban residence, healthcare access, and diagnostic-criteria change, and (vi) demonstrated interaction with genetic susceptibility. The first five overlap with conventional considerations used in GRADE and NHLBI assessments of observational evidence; gene–environment interaction was added because it is central to the hypothesis tested in this review. These parallels are heuristic rather than a claim of formal GRADE or NHLBI scoring. The rubric anchoring each grade is given in the footnote to **Table 1**, which also reports the key study design underpinning each grade so that the evidential basis is visible alongside the label.

**Table 1:** Strength of evidence for environmental determinants of multiple sclerosis.

Exposure	Representative effect estimate	Key study design	Epidemiological evidence	Biological plausibility	Principal limitation	Overall interpretation
EBV seroconversion	HR 32.4 (4.3–245.3) (5)	Nested case–control within >10 million-person military serial-serology cohort	Very strong (4, 5)	Very high	Near-universal exposure; single cohort for HR	Major established determinant; possibly necessary
Vitamin D/UV exposure	MR: OR 0.72 per SD 25OHD (0.60–0.87) (16)	Mendelian randomization, replicated; supporting observational cohorts	Strong; MR-supported (12, 13, 16, 17)	High	Latitude is a composite surrogate	Strong contributor; causality MR-supported
Adolescent obesity	MR: OR 1.33 per SD BMI (1.09–1.63) (16)	Mendelian randomization with mediation analysis	Strong; MR-supported (14–16)	High	Only ~5% mediated via vitamin D	Established; largely vitamin D-independent
Cigarette smoking	Observational consistent; MR null (OR 1.03, 0.77–1.40) (19)	Multiple case–control and cohort studies vs. two independent MR studies	Strong observational; MR discordant (8, 9, 19, 20)	Very high	MR–observational discordance; likely weak instruments	Established observationally; causal status less secure than vitamin D or BMI
Organic solvents	OR 1.50 (1.2–1.8) (43)	Population-based case–control (2,042 cases/2,947 controls) with HLA stratification	Moderate (43)	High	Binary self-reported exposure; single source population	Credible; strong HLA interaction, not independently replicated
Ambient PM_2.5_/PM_10_	HR 1.21 (1.07–1.38)/1.20 (1.02–1.42) (30)	Meta-analysis of 22 studies (16,585,206 participants), mixed cohort and case–control	Moderate (30, 34, 37, 38, 40)	High	*I*² = 95%; largest prospective cohorts null (32, 33)	Most credible emerging exposure; modest magnitude
Combustion-related NO*_x_*	OR 1.37 (1.10–1.76) (31)	Population-based case–control (6,635 cases/8,880 controls) with dispersion-modeled exposure	Moderate–strong (31)	High	Correlated pollutants; urban confounding; single source population	Dose–response and HLA synergy demonstrated
Occupational industrial dust	Reported association (44)	Population-based case–control with job-based exposure classification and HLA stratification	Moderate (44)	Moderate–high	Retrospective exposure classification	Promising; requires independent replication
Heavy metals	OR 2.03 (1.07–3.86) lead; 2.06 (1.08–3.91) mercury (47)	Case–control with self-reported exposure; meta-analyses of cross-sectional biomonitoring (16 and 58 studies)	Limited–moderate (47–50)	High	Cross-sectional biomonitoring; reverse causation; matrix–window mismatch	Causality unproven; large OR, weak design
PFAS/OH-PCBs	≈2-fold odds, highest vs. lowest (51)	Matched case–control (907 pairs) with mass-spectrometric serum measurement and mixture modeling	Limited (51)	Moderate–high	Serum sampled at diagnosis; single cohort	Emerging; mixture effect is the key finding
Diet and gut microbiome	Not quantified here (54)	Household-paired case–control (576 pairs, 7 centers); experimental transfer and dietary-intervention models (55–58)	Limited (52, 54)	Moderate–high	Temporality unresolved; therapy effects; no non-Western sites	Promising; pre-onset sampling required
Residential industrial proximity	SMR 1.31 (0.62–1.99) (45)	Community prevalence survey with distance-based exposure proxy	Weak/inconsistent (45, 46)	Moderate	Poor proxy for individual dose	Insufficient evidence
Gulf War deployment	RR 0.69 (0.61–0.78) (77)	Retrospective cohort of deployed vs. non-deployed personnel; registry interrupted time-series for civilians (79)	Contemporary evidence negative (77–79)	Theoretical	Heterogeneous exposure; secular trend	No convincing causal association

Rubric: Epidemiological evidence—very strong = ≥1 large prospective study with pre-onset exposure and consistent replication; strong = consistent observational evidence plus supportive Mendelian randomization; moderate–strong = large case–control or pooled evidence with dose–response and gene–environment interaction; moderate = ≥1 large well-designed study without independent replication; limited = cross-sectional or single-cohort evidence only; weak/inconsistent = conflicting results or null in the better-designed studies. Biological plausibility—very high = mechanism demonstrated in human disease; high = coherent mechanism with experimental support; moderate–high = plausible mechanism with limited direct evidence; moderate = general mechanism without specific evidence; theoretical = plausibility by analogy only. These grades derive from the six appraisal domains in Section 2.4, applied by consensus; they are structured expert judgment and are not GRADE certainty ratings (Section 2.4). Estimates are not directly comparable across rows (differing designs, contrasts, and effect measures).

MR, Mendelian randomization; HR, hazard ratio; OR, odds ratio; RR, relative risk; SMR, standardized morbidity ratio; SD, standard deviation; 25OHD, 25-hydroxyvitamin D; BMI, body mass index.

We do not label these grades as GRADE certainty ratings, and we do not report an inter-rater agreement statistic. Both authors considered the six domains for each exposure and reached final classifications by discussion against the primary sources; because grading was consensual rather than independently duplicated, any concordance statistic computed after the fact would misrepresent the process. Consequently, these grades are structured expert judgment; they are not reproducible in the sense that a duplicate-screened, instrument-scored appraisal would be, and they should be read as an ordering of evidential strength rather than as a measurement of it. Readers who require a reproducible appraisal of any single domain should consult the domain-specific systematic reviews cited throughout.

For the same reason, no PRISMA or PRISMA-ScR flow diagram is presented. The searches were not accompanied by duplicate screening with recorded record counts at each stage, and constructing a flow diagram retrospectively would present a reconstruction as a record. We state the absence explicitly here rather than substituting an approximation.

## 3 The evidential benchmark: established determinants

Emerging exposures should be judged against the standard set by established ones, and that standard is high. EBV seropositivity precedes MS in virtually all cases, seroconversion precedes neuroaxonal injury, and the effect size exceeds anything else in the field by an order of magnitude (4, 5). No emerging exposure approaches this (**Figure 2**).

**Figure 2:**
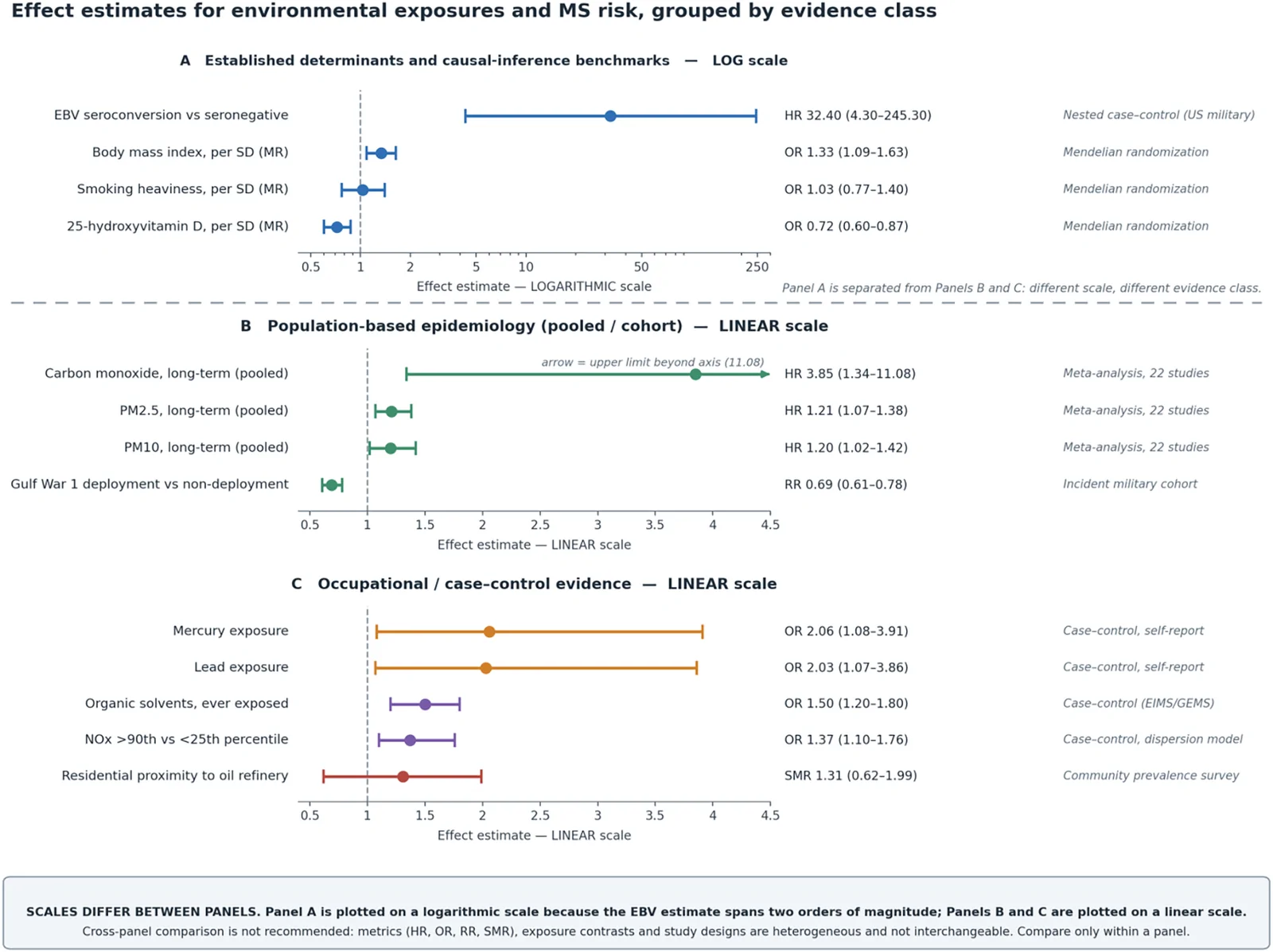
Effect estimates for environmental exposures and multiple sclerosis risk, grouped by evidence class. To avoid implying a single hierarchy of effect sizes across heterogeneous designs, estimates are separated into three panels: **(A)** established determinants and causal-inference benchmarks, including Mendelian-randomization estimates for body mass index, smoking heaviness, and 25-hydroxyvitamin D, **(B)** population-based pooled or cohort epidemiology, and **(C)** occupational and case–control evidence. Scales differ between panels and are labeled within each panel. **(A)** is plotted on a logarithmic scale because the EBV estimate spans two orders of magnitude, whereas **(B, C)** are plotted on a linear scale. **(A)** is separated from **(B, C)** by a horizontal rule to discourage direct comparison across different evidence classes and study designs. Comparison is appropriate within a panel, where designs are more similar, but is not recommended across panels since the metrics (HR, OR, RR, and SMR) and exposure contrasts differ and are not interchangeable. In **(B)**, an arrow indicates a confidence limit extending beyond the axis (carbon monoxide upper limit 11.08). The grouping makes the key dissociation legible: the largest point estimates among emerging exposures (metals, **C**) arise from self-reported case–control data, whereas the most robustly assessed exposures (particulate matter, **B**) show modest effects, and Mendelian randomization **(A)** supports vitamin D and body mass index while returning a null for smoking heaviness despite strong observational evidence. Estimates: EBV seroconversion (5); body mass index and 25-hydroxyvitamin D (16); smoking heaviness (19); carbon monoxide, PM_2.5_, and PM_10_ pooled (30); Gulf War deployment (77); mercury and lead (47); organic solvents (43); NO*_x_* (31); and refinery proximity (45).

Low vitamin D status and reduced early-life ultraviolet exposure are associated with susceptibility, though latitude is a composite surrogate rather than an isolated cause (6, 7). Here Mendelian randomization (MR) has been decisive: genetically decreased 25-hydroxyvitamin D is associated with increased MS odds (OR 0.72 per standard deviation increase, 95% CI 0.60–0.87) (12, 16), replicated independently (13, 17) and extended to pediatric-onset disease (14) and relapse hazard (18). Because genotype is fixed at conception, these designs are not vulnerable to the reverse causation that undermines observational vitamin D studies.

Adolescent obesity is supported on the same footing. Genetically increased body mass index is associated with increased MS risk (OR 1.33 per standard deviation, 95% CI 1.09–1.63) (15, 16), with concordant findings in pediatric-onset disease (14) and relapse-hazard analyses (18). Only approximately 5% of the obesity effect appears mediated through lowered vitamin D (16). This implies largely independent pathways, plausibly adipose-driven systemic inflammation, and implies that vitamin D supplementation would only modestly offset the effect of obesity.

Smoking is the most consequential case for this review, and the most awkward. Observational evidence is strong and consistent: smoking increases risk of onset (8, 21), worsens course, and interacts with HLA-DRB1*15:01 (3, 9), yet MR does not support it. Genetically predicted smoking heaviness was not associated with MS risk (OR 1.03, 95% CI 0.77–1.40), nor was smoking initiation, including multivariable models adjusting for body mass index (19); an independent MR study using a lifetime smoking index reached the same conclusion (20), and broader MR appraisals concur (23–25).

This discordance should neither be omitted nor over-read. One plausible explanation is limited validity and power of genetic instruments for cumulative inhaled tobacco exposure during the etiologically relevant window: available instruments proxy smoking initiation and heaviness, both behaviorally mediated traits, and MS genome-wide association sample sizes also constrain power. Observational studies additionally capture interaction with HLA that a main-effects MR analysis is not designed to detect. Residual confounding in observational studies remains an alternative explanation. The defensible position is, therefore, that smoking is a well-supported observational risk factor whose causal status is less secure than that of vitamin D or body mass index. This review treats it as a template for the inhalational hypothesis with that qualification attached, rather than as settled proof of it.

Alcohol shows an inverse association with MS risk in Swedish case–control data (22), a finding difficult to interpret and included here as a caution against reflexive causal reading of any single lifestyle correlate.

Two implications follow. First, studies of metals, pollution, or dust require explicit adjustment for smoking and correlated exposures; without it, an inhalational association is uninterpretable. Second, MR, triangulation across designs, and negative-control analyses should be the expected standard for any emerging determinant claiming causality—while the smoking case shows that a null MR result is itself evidence requiring interpretation rather than an automatic veto.

## 4 Ambient air pollution and combustion-related gases

Ambient air pollution is the emerging exposure with the most internally coherent evidence, though the coherence is more qualified than commonly presented. Unlike self-reported occupational history or post-diagnosis biomonitoring, pollution exposure can be estimated objectively through residential geocoding, monitoring networks, and dispersion modeling. This reduces recall bias and permits assessment of exposure preceding onset.

The most recent synthesis pooled 22 studies comprising 16,585,206 participants (30). Long-term exposure was associated with MS risk for PM_2.5_ (HR 1.21, 95% CI 1.07–1.38), PM_10_ (HR 1.20, 95% CI 1.02–1.42), and carbon monoxide (HR 3.85, 95% CI 1.34–11.08). Two features deserve emphasis and are usually omitted. First, the magnitudes for particulate matter are modest—roughly a 20% relative increase, far below EBV and comparable to many correlates of urban residence. Second, heterogeneity was substantial (*I*² = 95% for PM_2.5_). The pooled estimate, therefore, summarizes studies that disagree rather than converge, and the wide interval for carbon monoxide rests on few studies and should not be over-read.

The strongest single study remains a Swedish case–control analysis of 6,635 cases and 8,880 controls, with residential NO*_x_* estimated by spatially resolved dispersion modeling from 1990 onward (31). Exposure above the 90th percentile (24.6 µg/m³) versus below the 25th (5.9 µg/m³) yielded an OR of 1.37 (95% CI 1.10–1.76) with a significant trend across the exposure range (*p* < 0.0001). The investigators estimated that 13% of Swedish MS cases were attributable to NO*_x_* above the 75th percentile and reported synergy with HLA-DRB1*15:01. A parallel register analysis of 22,173 cases supported the association.

One caution should be registered here rather than deferred because it applies to every gene–environment interaction cited in this review. This finding, the organic solvent finding (Section 5), the industrial dust finding (Section 5), and the PFAS finding (Section 6) all derive from Swedish population-based case–control datasets (principally EIMS/GEMS) analyzed by overlapping research groups. They are, therefore, mutually consistent but not independently replicated, and their consistency partly reflects a shared source population and a shared analytic tradition. Sweden is also atypical in ways that bear directly on these exposures: it is a high-latitude population with low ambient ultraviolet exposure, a correspondingly distinct vitamin D distribution, a comparatively narrow ambient pollution range, and a northern European HLA allele distribution in which DRB1*15:01 is common. Whether the interaction generalizes beyond this setting is, at present, unknown. Section 10 argues that this is the single most valuable thing an external population could establish.

Against the Swedish findings, the two largest prospective cohorts were null. In the Nurses’ Health Study and NHS II, particulate exposure was not associated with MS risk (32); in an Ontario population-based cohort, the estimate was similarly unrevealing (33). This discrepancy is the central interpretive problem in the field and is rarely stated plainly. Positive findings cluster in case–control designs with retrospective residential reconstruction, while prospective cohorts with pre-onset exposure assignment have not replicated them. The optimistic reading is that dispersion modeling across decades captures the relevant etiological window better than cohort-era address data. The skeptical reading is that case–control designs are more permeable to residual confounding by urban residence and socioeconomic position. Both readings are currently tenable, and the field should say so.

Evidence for disease activity, as opposed to susceptibility, is somewhat more consistent: particulate exposure has been associated with inflammatory activity on MRI (34), with relapse (35, 36), with pediatric MS (37), and with prevalence gradients in northern Italy (38); broader reviews reach similar conclusions (39). An ecological comparison from Türkiye between the steel-producing city of Ereğli and the less polluted Devrek reported more than twofold higher MS prevalence in the industrial setting (40). The design cannot support causal inference, but it did stimulate the field.

Mechanistically, inhaled particles generate pulmonary and systemic inflammation, oxidative stress, endothelial dysfunction, and innate immune activation, and ultrafine fractions may translocate across biological barriers. The most specific supporting observation is that autoreactive T cells acquire a migratory phenotype licensing central nervous system entry within the lung (41). This supplies a concrete route from an inhaled exposure to a central autoimmune outcome; pollutant-associated changes in immune activity have also been reported in MS cohorts (42). Section 8 develops this into a specified molecular account.

Limitations remain material. Pollutants are correlated mixtures, so isolating a causal component is generally not possible with observational data. Residential estimates miss occupational, commuting, and indoor exposure. Residual confounding by urban residence persists. The honest summary is that ambient air pollution is the most credible emerging determinant of MS while remaining unproven and that its plausible effect size is modest.

## 5 Occupational and industrial inhalational exposures

If the inhalational hypothesis is correct, occupational exposure should show a stronger signal than residential proximity because intensity and duration are far greater and exposure is less confounded by the broad correlates of urban living. This prediction is broadly borne out, with the replication caveat set out in Section 4 applying throughout.

Organic solvents are the most instructive case and are frequently omitted from environmental MS reviews. In a Swedish population-based case–control study of 2,042 incident cases and 2,947 controls, ever-exposure carried an OR of 1.50 (95% CI 1.2–1.8, *p* = 0.0004) (43). More importantly, the effect was concentrated in carriers of HLA-DRB1*15 lacking HLA-A*02, and combined with smoking the risk in that genotype stratum rose approximately 30-fold relative to unexposed non-carriers. This shows a smoking–HLA interaction pattern with a different agent—but, as noted, in the same population.

Occupational industrial dust shows a similar architecture. A Swedish population-based case–control study reported an association with incident MS, amplified by smoking and by HLA-DRB1*15:01 (44), mirroring the pattern seen for combustion-related pollution (31). The recurrence of a broadly similar genotype-restricted risk pattern across three chemically distinct inhaled agents—tobacco smoke, solvents, and mineral dust—is, in our view, among the more suggestive observations in the emerging-exposure literature. Two qualifications attach to it. First, these studies draw on related Swedish datasets and research groups, so they do not constitute fully independent replications across unrelated populations (Section 4). Second, interaction estimates of this kind are sensitive to model specification and to the choice of additive or multiplicative scale. It is nonetheless notable that the convergence is visible in interaction analyses even where main-effect Mendelian randomization is null (Section 3), which is consistent with exposures acting principally within a susceptible genotype stratum.

Residential proximity to industry performs considerably worse. A US investigation prompted by community concern examined MS prevalence around a closed oil refinery: age-adjusted period prevalence across the whole study area was 113 per 100,000 (95% CI 93–136), and for the community bordering the refinery specifically the standardized morbidity ratio was 130.8 (95% CI 62.3–199.3), compatible with no excess (45). A related three-community investigation found that prevalence tracked latitude and ultraviolet exposure more closely than industrial proximity (46). Residential distance is a poor proxy for personal dose, varying with meteorology, terrain, tenure, occupation, and behavior.

Occupational studies retain their own vulnerabilities: retrospective exposure classification, smoking confounding, socioeconomic gradients, and simultaneous exposure to multiple agents. Job-exposure matrices and quantitative reconstruction, rather than binary ever/never self-report, are the necessary next step.

## 6 Heavy metals and persistent organic pollutants

Heavy metals—principally lead, mercury, cadmium, and arsenic—have long been candidate MS toxicants. Plausibility derives from oxidative stress, mitochondrial impairment, immune dysregulation, and blood–brain barrier compromise (39, 47). Epidemiological support is considerably weaker than for inhalational exposures.

A population-based case–control study found that MS cases are more likely than matched controls to report occupational or environmental lead exposure (OR 2.03, 95% CI 1.07–3.86) and mercury exposure (OR 2.06, 95% CI 1.08–3.91), with exploratory gene–environment analyses (47). A meta-analysis of 16 studies (772 cases and 878 controls) reported heterogeneous but generally elevated arsenic, lead, mercury, and cadmium concentrations in MS (48). A larger synthesis of 58 studies and more than 10,000 participants found higher arsenic and cadmium but no consistent differences for several other metals, with marked variability across countries and methods (49). A US ecological analysis of MS prevalence and possible lead exposure was likewise inconclusive (50).

These findings are suggestive but do not establish causality, and the reason is structural rather than incidental. Most biomonitoring is cross-sectional and measures concentrations after onset. Disease-related changes in mobility, diet, occupation, medication, and physiology can alter measured concentrations independently of etiological exposure. Reverse causation is, therefore, not a peripheral caveat but the dominant alternative explanation.

### 6.1 Windows of susceptibility and the matrix problem

A second structural problem is specific to metals and is under-discussed. Biological half-lives differ by orders of magnitude between metals and between compartments of the same metal. Lead is retained in bone for years to decades and is mobilized during periods of bone turnover; blood lead, therefore, reflects both recent intake and endogenous release from a long-term skeletal store. Inorganic mercury and methylmercury clear from blood over weeks to months, so a blood concentration indexes recent intake almost exclusively. Cadmium accumulates in the renal cortex over decades, while urinary cadmium reflects that accumulated body burden and blood cadmium reflects recent exposure. Arsenic is excreted in urine within days, so urinary arsenic is a short-window measure unless speciated and repeated.

Two consequences follow. First, each matrix answers a different question: hair and nail index intermediate windows, blood indexes recent intake for some metals and lifetime burden for others, and urine indexes excretion over days. A study measuring blood mercury and a study measuring urinary cadmium are not measuring the same construct, and pooling them assumes an equivalence that does not hold. This is a substantial and probably under-appreciated source of the between-study heterogeneity in the metals meta-analyses (48, 49). Second, and more importantly for etiology, the exposure window that matters for MS is likely to be childhood, adolescence, and early adulthood (Section 8.6), whereas almost all available measurements are taken in adults at or after diagnosis. For metals with short biological half-lives, a post-diagnosis measurement carries essentially no information about the etiologically relevant window. Only metals with long-term storage compartments—principally lead in bone, measurable by K-shell X-ray fluorescence rather than by blood sampling—retain a signal from the relevant period. Future studies should state which window a chosen matrix indexes and should justify that choice against the hypothesized etiological window rather than against convenience.

Note also the design asymmetry visible in **Figure 2**: the metals estimates are numerically larger than those for air pollution but derive from self-reported exposure in case–control studies. Effect size should not be mistaken for evidential strength.

### 6.2 Persistent organic pollutants

Persistent organic pollutants are a more recent and more methodologically interesting entrant. In 907 MS cases and 907 matched controls from the Swedish EIMS cohort, serum concentrations of 14 per- and polyfluoroalkyl substances (PFAS) and three hydroxylated polychlorinated biphenyls (OH-PCBs) were measured by mass spectrometry (51). Several compounds—notably perfluorooctane sulfonic acid, 4-OH-CB187, and 3-OH-CB153—showed non-linear associations with MS odds, with roughly twofold odds at the highest versus lowest concentrations.

Two results matter more than the point estimates. First, mixture modeling showed that combined PFAS and OH-PCB exposure raised MS odds even where individual compounds showed weak or absent effects—a direct empirical demonstration of the exposome’s core claim that single-agent analysis under-detects real risk. Second, the PFOS association was modified by HLA-B*44:02, extending gene–environment interaction beyond the DRB1 locus. The same caveats apply as for metals, with one addition: serum was measured at or near diagnosis, so temporality is not established; the study is best read as a strong rationale for pre-onset biobanked measurement rather than as evidence of causation; and it derives from the same Swedish source population as the solvent and dust findings (Section 4).

From an exposome perspective, metals and persistent pollutants are difficult to isolate precisely because they co-occur with smoking, occupational dust, industrial emissions, and ambient particulate matter—and, as Section 10 argues, in some regions they are delivered in the same inhaled particle.

## 7 Diet, obesity, and the gut microbiome

Diet and the gut microbiome are the internal face of the exposome and are frequently treated as a separate literature. The separation is artificial, since ingested exposure is exposure.

This section is deliberately selective, and the selection rule is stated so that it can be assessed. The dietary and microbiome literature in MS is large, heterogeneous, and dominated by cross-sectional and short-term interventional work; surveying it would not change the appraisal in **Table 1** because almost none of it meets the temporality criterion. We, therefore, cite (i) the exposure with Mendelian randomization support (obesity), (ii) the largest controlled human microbiome comparison, (iii) experimental work that specifies a molecular node used in the working model of Section 8, and (iv) studies that establish or contest the direction of the diet–immunity relationship at a mechanistic level. Studies that report compositional differences without addressing temporality or mechanism are not individually cited; readers are referred to the domain-specific reviews (52, 53).

Obesity in adolescence is the best-established dietary-adjacent determinant and is supported by Mendelian randomization (15, 16), as set out in Section 3. Because only a small fraction of its effect appears mediated by vitamin D, adipose-driven systemic inflammation is the leading candidate pathway—the same final common pathway implicated in inhalational exposure.

The microbiome hypothesis holds that environmental exposure is partly transduced through gut microbial communities that shape systemic immune tone. The largest controlled assessment to date, the International MS Microbiome Study, compared 576 MS patients with genetically unrelated household controls (1,152 subjects in total), a design that substantially reduces confounding by diet, geography, and shared environment, and identified associations with both disease risk and course (54). Household pairing matters: much earlier microbiome work compared cases with unmatched controls and could not separate microbial signal from lifestyle. Two limitations of this study should be stated alongside its strengths. The cohort, while the largest of its kind, remains modest for a compositional analysis with many features and many potential confounders. More importantly, recruitment was from seven centers in North America, Europe, and South America; there are no Middle Eastern, African, or Asian sites. Since the gut microbiome varies strongly with dietary pattern and geography, generalization of specific taxonomic associations to populations with different habitual diets—including those of the Gulf region—is not warranted, and regional replication is needed before any taxon is treated as a general MS marker.

Reviews have appropriately emphasized that composition differences may follow disease, treatment, or diet rather than precede them (52). Disease-modifying therapy, altered mobility, and dietary change after diagnosis all plausibly reshape the microbiome, so cross-sectional case–control comparison faces the same reverse-causation problem that limits metals biomonitoring.

Experimental work has, however, moved beyond composition to mechanism, and four lines are directly relevant to the working model. First, intermittent fasting ameliorated the clinical course and pathology of experimental autoimmune encephalomyelitis (EAE), increased gut microbial richness, and—critically for causal interpretation—conferred protection when microbiota from fasted animals were transferred into recipients; partially concordant microbiome changes were observed in a small parallel study of intermittent energy restriction in relapsing MS (55). Fecal transfer conferring the phenotype is the design feature that distinguishes a microbiome-mediated effect from a microbiome correlate. Second, a diet-dependent host metabolite has been shown to shape the gut microbiota in a manner protective against autoimmunity: ketogenic-diet-derived β-hydroxybutyrate acted on the microbiota to reduce disease severity in EAE, identifying a host-metabolite-to-microbe direction of effect rather than the usual reverse (56). Third, macronutrient composition itself—rather than any single nutrient—maps onto disease burden: nutritional-geometry analysis of global supply data found higher carbohydrate supply associated with greater MS burden and higher fat supply with less, and an isocaloric high-fat, low-carbohydrate diet was protective in EAE with transcriptional and epigenetic pre-programming of naïve T cells toward a less activated phenotype (57). Ecological macronutrient-supply analyses are of course confounded by economic development, and the authors are appropriately cautious; the value of the study lies in the concordance between the epidemiological signal and the experimental one. Fourth, the causal architecture linking Th17 responses to the gut has been directly interrogated: in IL-17A/F-deficient mice, susceptibility to EAE was restored by cohousing, by fecal transfer, and by gut-restricted re-expression of IL-17A, indicating that a substantial part of IL-17’s effect on CNS autoimmunity is exerted through the intestinal microbiome rather than within the CNS (58). This last observation is important for the model developed in Section 8 because it places the Th17 axis and the microbiome on the same pathway rather than in parallel.

Taken together, these studies establish that diet–microbiome effects on CNS autoimmunity are, in experimental systems, transferable, metabolite-mediated, and macronutrient-sensitive. What they do not establish is that the same holds in humans at ambient levels of dietary variation. No dietary factor besides obesity currently meets the evidential standard set out in Section 3. Prospective, pre-onset sampling with paired controls, with serum and fecal metabolite measurement rather than composition alone, is the design that would change this.

## 8 Immunological mechanisms: how environmental exposures reshape effector–regulatory balance

The evidence reviewed above is epidemiological. This section sets out the mechanistic claim of the working model—claim 2 of Section 1.2—and it precedes the regional argument deliberately because a regional test is only worth proposing if it can be tied to specified molecular events and measurable immunological endpoints.

One important axis in MS immunopathology is the imbalance between pro-inflammatory effector T-cell responses—principally IL-17-producing T helper 17 (Th17) and IFN-γ-producing Th1 populations—and regulatory T-cell (Treg) function, operating within a broader network involving autoreactive B cells, CD8^+^ T cells, innate immune cells, and CNS-resident inflammatory responses (59). Contemporary MS immunology is not reducible to a single effector–regulatory axis: the efficacy of B-cell-depleting therapy, the role of B cells as antigen-presenting cells, CD8^+^ T-cell-mediated oligodendrocyte injury, and compartmentalized inflammation sustained by microglia and CNS-resident macrophages are all central (59, 60). A mechanistic account of the exposome need not explain all of these, but it must at least specify how chemically diverse environmental agents perturb identifiable nodes within this network. Four largely independent molecular routes do so, with several converging toward the effector–regulatory balance. Their convergence—rather than any single pathway—is what gives the exposome framework immunological substance (**Figure 3**).

**Figure 3:**
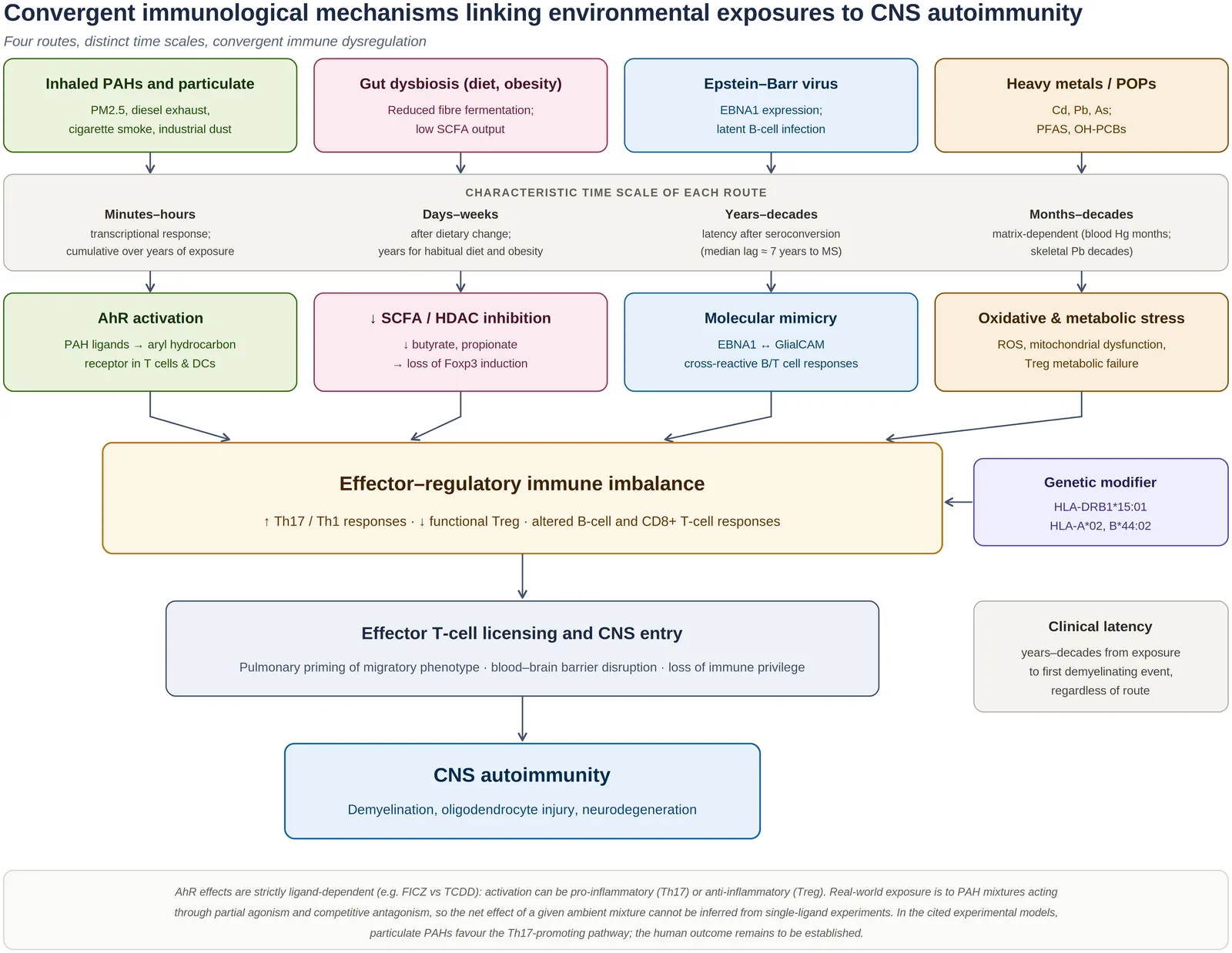
Convergent immunological mechanisms linking environmental exposures to CNS autoimmunity, with the characteristic time scale of each route. Four largely independent molecular routes converge toward effector–regulatory immune imbalance—increased Th17/Th1 responses, reduced functional Treg, and altered B-cell and CD8^+^ T-cell responses—within the broader immune network implicated in MS. The routes operate on very different time scales, shown in the central band: AhR-mediated transcriptional responses occur within minutes to hours of exposure but require chronic repetition (62, 63, 65); SCFA availability changes within days to weeks of a dietary change and over years with habitual diet and obesity (68, 70); EBV-associated mechanisms unfold over a latency of years to decades, with a median lag of roughly 7 years from seroconversion to MS (5, 60, 72); and metal-associated oxidative burden accumulates over months to decades depending on the storage compartment (47, 70). Inhaled polycyclic aromatic hydrocarbons and particulate can activate the aryl hydrocarbon receptor (AhR) in T cells and dendritic cells; in specific experimental systems, this enhances Th17 polarization, though AhR effects are strictly ligand-dependent and can also be Treg-promoting (62, 63, 65). Gut dysbiosis reduces short-chain fatty acid (SCFA) output, lowering histone deacetylase inhibition and Foxp3^+^ Treg induction (58, 68, 70). Epstein–Barr virus may provide an antigenic trigger, in part through EBNA1/GlialCAM molecular mimicry and in part through other B-cell-centered mechanisms (60, 72). Heavy metals and persistent organic pollutants generate oxidative and metabolic stress that may disproportionately impair regulatory T-cell function, a mechanism requiring human validation (47, 70). The resulting effector-skewed responses are influenced by HLA-restricted genetic susceptibility (class I and class II alleles), may be licensed to a migratory phenotype in the lung, and traffic across a disrupted blood–brain barrier (41, 73). Clinical latency from exposure to first demyelinating event is years to decades regardless of route. Directionality is ligand-dependent: environmental PAHs favor the Th17-promoting pathway in the cited experimental models, whereas the synthetic ligand TCDD is Treg-promoting (63); real-world exposure is to PAH mixtures acting through partial agonism and competitive antagonism, so the net effect of an ambient mixture cannot be inferred from single-ligand experiments (Section 8.1).

### 8.1 The aryl hydrocarbon receptor: a direct sensor of inhaled pollutants

The strongest mechanistic bridge between air pollution and CNS autoimmunity is the aryl hydrocarbon receptor (AhR), a ligand-activated transcription factor for which polycyclic aromatic hydrocarbons (PAHs), dioxins, and polychlorinated biphenyls are high-affinity ligands (61). Two landmark studies established that AhR controls the Th17/Treg decision in a strictly ligand-dependent manner: activation by the endogenous ligand FICZ boosts Th17 differentiation and worsens EAE, whereas activation by the synthetic ligand TCDD expands Foxp3^+^ Treg cells and suppresses EAE (62, 63).

This ligand dependence is the central caveat as much as the central mechanism. AhR activation is not intrinsically pro-inflammatory, and the immunological outcome of a given exposure cannot be inferred from AhR engagement alone. The point requires strengthening for real-world exposure. Ambient particulate delivers not a single ligand but a complex PAH mixture, together with nitro- and oxy-PAH derivatives, transition metals, and endotoxin. Within such mixtures, individual constituents differ in affinity, in the duration of receptor occupancy, and in whether they act as full agonists, partial agonists, or competitive antagonists; rapid CYP1A1-mediated metabolism of some ligands further shortens signaling and can convert an apparent agonist into a transient one. The net transcriptional output of an ambient mixture is, therefore, not the sum of its single-ligand effects and cannot be predicted from them. Readers should not take from this section the impression that inhaled PAHs are necessarily pro-inflammatory in humans.

What is relevant here is the narrower empirical observation, in specific experimental systems, that atmospheric particulate matter and diesel exhaust particles can enhance Th17 polarization in an AhR-dependent fashion in both cell culture and murine models, an effect abolished in AhR-deficient cells and attributed to the PAH fraction (64–66). These findings suggest a plausible molecular route by which some inhaled particulate could raise the frequency of the effector T-cell subset implicated in demyelination—a route the epidemiological literature otherwise lacks. They do not establish that environmental PAHs uniformly produce a Th17-promoting outcome in humans. This possibility connects to Section 10 since the Riyadh particulate burden is PAH-bearing (67), and Section 10.6 specifies what would have to be measured to test it, including the mixture-level ambiguity described here.

### 8.2 Short-chain fatty acids: the metabolic link between diet, microbiome, and tolerance

The second route runs through the gut. Microbial fermentation of dietary fiber yields short-chain fatty acids (SCFAs)—acetate, propionate, and butyrate—that act both as histone deacetylase inhibitors and through the G-protein-coupled receptors GPR43, GPR41, and GPR109A to promote Foxp3^+^ Treg differentiation and suppress Th17 and Th1 responses (68, 69). This is not merely an animal-model observation. Serum and fecal propionate are significantly reduced in people with MS, particularly after the first relapse. In an open-label proof-of-concept study, propionate supplementation added to immunotherapy was associated with a sustained increase in functionally competent Treg cells and a decrease in Th17 and Th1 cells, together with normalization of Treg mitochondrial function (70).

In exploratory retrospective longitudinal observations from the same cohort, up to 3 years of intake was associated with lower annual relapse rate, disability stabilization, and reduced brain atrophy. These were not endpoints of a randomized controlled trial and should be read as hypothesis-generating rather than as established intervention efficacy (70). SCFAs additionally reinforce blood–brain barrier integrity by upregulating endothelial tight-junction proteins (71). Dysbiosis driven by low-fiber diet and by obesity—itself an MR-supported MS determinant (Section 3)—thus plausibly lowers a tonic Treg-inducing signal, shifting the effector–regulatory balance in a direction opposite to that of AhR activation by particulate PAHs. The gut-restricted IL-17 experiments discussed in Section 7 (58) place the Th17 arm of this axis, too, partly within the intestine.

### 8.3 Molecular mimicry and other EBV mechanisms

The third route offers a molecular account of the field’s strongest determinant. High-affinity molecular mimicry has been demonstrated between the EBV transcription factor EBNA1 and the CNS glial protein GlialCAM: clonally expanded B cells in MS cerebrospinal fluid bind both, EBNA1 immunization exacerbates EAE, and anti-EBNA1/anti-GlialCAM antibodies are prevalent in patients (60). EBV may, therefore, provide an antigenic trigger in part through EBNA1/GlialCAM mimicry.

Mimicry is, however, one proposed mechanistic bridge rather than the settled explanation of EBV’s relationship to MS. EBV-infected B cells may also contribute through aberrant antigen presentation, provision of survival signals to autoreactive lymphocytes, and dysregulated cytotoxic control; impaired CD8^+^ T-cell control of EBV-induced autoimmunity independently raises MS risk (72). For an exposome argument, the salient point is not which EBV mechanism dominates, but that EBV supplies an antigenic and B-cell-centered substrate upon which environmental modifiers act. Whichever bridge operates, AhR activation, SCFA depletion, and genetic background plausibly set the effector-versus-regulatory threshold at which the EBV-associated response provokes disease.

### 8.4 Oxidative stress, metabolic failure, and the lung–CNS axis

The fourth route is the least specific but unifies the toxicant domains. Heavy metals and persistent organic pollutants promote reactive oxygen species generation and mitochondrial dysfunction (39, 47), and Treg suppressive function is critically dependent on oxidative mitochondrial metabolism—the same metabolic axis whose normalization accompanied propionate supplementation (70). Metabolic stress may, therefore, disproportionately impair regulatory immune function. This is a plausible mechanism requiring direct validation in human exposure studies, not one demonstrated for metals or persistent pollutants in human MS.

Overlaying the toxicant routes is an anatomical one: inhaled particulates initiate pulmonary and systemic inflammation (9, 31, 43, 44), autoreactive T cells can acquire within the lung a migratory program that licenses CNS entry (41), and barrier disruption permits chemokine-directed trafficking of primed effector cells across the blood–brain barrier (73). This supplies a specified anatomical route that a purely systemic metals hypothesis lacks.

### 8.5 Gene–environment interaction across the routes

The recurrence of HLA-restricted interaction supports, without proving, a shared susceptibility framework. The pattern appears across several chemically distinct exposures: combustion-related NO*_x_* with HLA-DRB1*15:01 (31); occupational dust with the same allele and with smoking (44); organic solvents with HLA-DRB1*15 in the absence of HLA-A*02, amplified by smoking (43); PFOS with HLA-B*44:02 (51); and head trauma with MS-associated HLA genes (74).

These interactions involve both HLA class II (DRB1*15:01) and HLA class I (A*02, B*44:02) alleles, so the shared susceptibility they imply is not reducible to a single antigen-presentation pathway. For the class II interactions, the observation is mechanistically coherent: because HLA-DRB1 determines which peptides—potentially including the EBNA1/GlialCAM mimics of Section 8.3—are presented to CD4^+^ T cells, a DRB1-restricted architecture is consistent with a mechanism operating through antigen-specific effector T-cell differentiation. The class I interactions (A*02, B*44:02) more plausibly act through CD8^+^ T-cell recognition or NK-cell regulation and should not be interpreted through the class II presentation model.

Two cautions, already introduced in Section 4, apply with full force here. First, these observations derive from related Swedish population-based datasets and research groups, so they are not fully independent replications across unrelated populations. Second, interaction estimates of this kind are sensitive to model specification. The recurrence of a broadly similar HLA-restricted interaction pattern across chemically distinct inhalational exposures is, therefore, noteworthy and is consistent with a shared susceptibility framework (3, 59), though it does not establish one. Testing it in a genetically and geographically distinct population, as Section 10 proposes, is the natural next step, and Section 10.2 sets out why the HLA composition of Gulf populations makes that test informative rather than merely additional.

### 8.6 Critical windows

Timing may matter as much as agent. Recent systematic evidence highlights childhood and adolescence as periods of susceptibility (11), strongest for established factors—ultraviolet exposure, vitamin D status, and adolescent body mass index (6, 7, 14, 15)—with migration studies indicating that early-life environmental context shapes later risk (53). The regional finding that school-age and university-age sun exposure is protective is consistent with this (75).

Whether the emerging exposures share these windows is unknown, because essentially all positive studies of pollution, dust, and solvents assess adult or lifetime exposure. The four mechanistic routes also operate on very different time scales, which **Figure 3** makes explicit: AhR transcriptional responses occur within minutes to hours of exposure but require chronic repetition to matter; SCFA availability changes within days to weeks of a dietary change but reflects habitual diet over years; EBV-associated mechanisms unfold over a latency of years to decades, with a median lag of roughly 7 years from seroconversion to MS (5); and metal-associated oxidative burden accumulates over months to decades depending on the storage compartment (Section 6.1). Mechanisms operating on such different time scales cannot share a single critical window, and a study designed around one of them will be mis-specified for the others. Epigenetic regulation—including the HDAC inhibition through which SCFAs act (68, 70)—and microbiome-mediated immune programming (52, 54) are plausible mediators of window effects but require dedicated human study.

## 9 Measuring the exposome: technical frontiers and what this review assumes

A review with “exposome” in its title should be explicit about which version of the concept it applies because the term now covers two quite different research programs.

The first is the classical external–internal accumulation framework, in which named exposures are measured individually across the life course and combined (26, 27). The second is the agnostic, discovery-oriented program in which the internal chemical environment is characterized without a prior hypothesis, typically by high-resolution mass spectrometry, and associations with disease are sought across the resulting feature space (76). This review adopts the first framework. Every exposure appraised in Sections 3–7 is a named, hypothesis-led exposure with a prior literature, and the appraisal criteria in Section 2.4 presuppose that a specific exposure–outcome contrast exists. Readers should, therefore, not take this review as a survey of MS exposomics in the second sense, which barely exists.

That gap is itself worth stating because it is the main reason the MS exposome remains a framework rather than a measurement program. Four technical developments would change this.

High-resolution untargeted exposomics: High-resolution mass spectrometry combined with cheminformatic annotation now permits simultaneous detection of hundreds to thousands of exogenous chemicals and their metabolites in a single biospecimen, and network-based approaches allow structure to be recovered from the resulting feature space (76). The PFAS/OH-PCB study discussed in Section 6.2 is a targeted precursor of this approach: it measured 17 pre-specified compounds and found that the mixture carried signal the individual compounds did not (51). An untargeted analysis of the same specimens would not have required the compounds to be nominated in advance.

Exposome-wide association analysis: The analytic counterpart is to treat measured exposures as a high-dimensional predictor set and to test them against disease with appropriate control of multiplicity and correlation structure, as genome-wide association analysis does for genotype. A measured-exposure exposome-wide association study (ExWAS) has not yet become an established part of the MS epidemiological literature. Its main requirements—large numbers of cases with uniformly collected biospecimens and a defined source population from which controls can be sampled—are registry requirements as much as laboratory ones.

Personal-level exposure measurement: Residential or workplace geocoding assigns a modeled ambient concentration to a person, which is not the same as the dose that person receives. Personal monitoring, wearable and passive samplers, time-activity reconstruction, and mobile-phone-derived location traces bring measurement closer to individual dose and, importantly, capture indoor, commuting, and occupational microenvironments that dispersion models miss. In settings where a large fraction of the day is spent in air-conditioned indoor environments—as is the case across the Gulf for much of the year—the divergence between modeled ambient exposure and personal dose may be particularly large, and this is measurable rather than merely arguable.

Integration with immunophenotype: The step that would make exposome data mechanistically interpretable is the pairing of exposure measurement with immunological read-out in the same individuals: Th17 and Treg frequency and function, Treg mitochondrial respiration, serum and fecal SCFAs, AhR target-gene induction (CYP1A1/CYP1B1) in circulating mononuclear cells, EBV serology, and neuroaxonal injury markers such as serum neurofilament light chain and GFAP. An exposome–immunophenotype design of this kind converts an epidemiological association into a testable mechanistic chain, and Section 10.6 specifies one.

Implementing any of this outside high-income European and North American settings raises practical obstacles that are rarely acknowledged in reviews written from within those settings: biobank construction with pre-analytic standardization adequate for trace chemical analysis; harmonized consent and data-governance frameworks permitting linkage between clinical registries, environmental monitoring authorities, and laboratories; local high-resolution mass spectrometry capacity and reference libraries; and agreed data standards allowing regional cohorts to be pooled with international ones. Several of these are institutional rather than conceptual scientific barriers and are potentially tractable through coordinated governance, standardization, and investment. Section 10.4 identifies which components already exist in the Gulf setting and which would need to be built.

## 10 The Gulf exposome: a proposed natural test case

Sections 3–7 established what the evidence supports, Section 8 specified the mechanism, and Section 9 specified what would have to be measured. This section applies all three to a setting and states in advance what result would count against the model.

### 10.1 Clearing the ground: what the Gulf War evidence does and does not show

The regional argument must first be separated from a claim that is frequently attached to it. The proposition that the 1990–1991 Gulf War increased MS risk—attributed to oil well fires, combustion products, chemical exposures, and stress—requires separating two questions: whether military deployment increased risk and whether conflict-related environmental disruption altered incidence among resident civilians. Neither is supported by contemporary data.

On deployment, large US Department of Defense and Veterans Affairs cohorts found no increased risk among personnel deployed during the first Gulf War relative to non-deployed personnel of the same era: from 1,841 incident cases, the relative risk for MS was 0.69 (95% CI 0.61–0.78), non-significant or apparently protective across race, age, and service branch (77, 78). A healthy-warrior effect plausibly contributes to estimates below unity, but the finding does not support deployment as a risk factor. On civilian incidence, an interrupted time-series analysis of 1,764 incident cases in the Kuwait National MS Registry (1980–2019) found incidence already rising before the Iraqi invasion, with neither the invasion-period step change nor the pre- versus post-war trend difference reaching significance; the rise was attributed to improved diagnostics, expanding services, and registry development (79). Historical reports of postwar MS “epidemics,” notably the Faroe Islands (80, 81), predated modern diagnostic criteria and MRI-based ascertainment and remain speculative.

Armed conflict plausibly generates particulate, metals, combustion products, and healthcare disruption, but plausibility is not evidence. This negative finding is useful here for two reasons. It clears the ground for a question that concerns the present-day exposome of the region rather than the war, and it demonstrates that regional registry data are already capable of testing a specific environmental hypothesis and returning a null result—which is the relevant evidence that the infrastructure argued for below can, in fact, discriminate.

### 10.2 Why the Gulf Cooperation Council region and not another high-exposure setting

The Gulf is not the only region with high particulate exposure and rising MS occurrence. Northern India, northern China, and Mexico City all have ambient concentrations comparable to or exceeding those of the Gulf, and all have reported increasing MS recognition. The argument for the Gulf is, therefore, not that its air is uniquely polluted. It is that four conditions coincide there, and their conjunction is unusual.

Existing national case ascertainment: Testing an exposure–response relationship requires a defined source population, complete or near-complete case capture, and a sampling frame for controls. National MS registries operate in Saudi Arabia and Kuwait (79, 82, 83), and the Kuwaiti registry has already supported a formal quasi-experimental analysis (79). Registry coverage of this kind is the binding constraint in most high-pollution settings, and it is the constraint that determines whether a study is a case–control analysis or an ecological one.

Potentially reconstructable residential histories in citizen populations: Long-window exposure reconstruction is feasible only if residential histories are measured rather than assumed. Citizen populations may offer longer within-country residence than large expatriate groups, but internal mobility remains possible and must be captured explicitly. Expatriate populations have shorter and more heterogeneous residence histories and should, therefore, be analyzed separately, or excluded from long-latency analyses unless pre-migration exposure can be reconstructed. This is a design opportunity, not an assumption of immobility.

Existing environmental measurement: Long-term multi-site ambient monitoring and speciated particulate composition data already exist for at least one major city (67, 84, 85). The value of speciation is developed in Section 10.3; without it, a study can relate MS to mass concentration only, which is the least informative version of the question.

A genuinely different genetic background: This is the least obvious and possibly the most important condition. In the Middle East and North Africa region, HLA-DRB1*15 is associated with MS, but the association is weaker than in northern European populations, and other alleles carry risk (DRB1*03 and DRB1*04) or apparent protection (DRB1*07 and DRB1*11) (86). Population-level HLA data from 28,927 Saudi stem-cell donors show an allele distribution differing substantially from northern European reference populations, with DRB1*07:01 and DRB1*03:01 as the most frequent DRB1 alleles (87). This matters for the specific claim at issue. The gene–environment interactions in Section 8.5 were characterized in a population where DRB1*15:01 is common and the surrounding haplotype is relatively homogeneous. A replication in a population where the allele is less frequent, sits on different haplotypes, and coexists with different candidate risk alleles is not a repetition of the Swedish result but a test of whether the interaction is a property of the allele and the exposure or a property of the Swedish population and its correlated exposures. That distinction cannot be resolved by another study within northern Europe.

The corresponding disadvantages should be stated. MS incidence data across the region are sparser than prevalence data, genotyping capacity exists in tertiary centers but is not organized as a research resource, and no GCC country currently has a population biobank with pre-onset serum linked to a clinical registry. The proposal below is, therefore, for a design that uses what exists and identifies separately what would need to be built.

### 10.3 The exposure: extreme, characterized, and compositionally distinctive

Two features of Gulf particulate matter shape everything that follows, and the second is often treated as a caveat when it is better understood as the analytical opportunity.

Magnitude: A 9-year assessment across 13 fixed monitoring stations in Riyadh found the citywide annual mean PM_2.5_, PM_10_, and NO_2_ to be above WHO Air Quality Guideline values in every monitored year from 2015 to 2023, with particulate matter being the dominant driver of air quality index and marked spatial heterogeneity across districts (84). Trends diverged by pollutant—declining PM_2.5_, PM_10_, and CO alongside rising NO_2_ and O_3_. Divergent trends are analytically valuable in themselves since they permit partial separation of correlated pollutants that move together in European settings. Crucially, the exposure contrast lies far above the Swedish range in which the NO*_x_* dose–response was characterized (31), where the 90th percentile was 24.6 µg/m³. If the dose–response is real and monotonic, it should be detectable here; if the Swedish finding is confounded by urban socioeconomic position, the Gulf offers a partly decoupled test because the dominant particulate source is regional desert dust rather than urban traffic density.

Composition: Speciation of Riyadh PM found a mean PM_10_ value of 223.12 ± 66.12 µg/m³ against PM_2.5_ of 35.49 ± 9.63 µg/m³—consistent with a dominant local dust source—and quantified iron, nickel, chromium, zinc, cobalt, copper, silver, arsenic, cadmium, and lead within both fractions, with enrichment analysis indicating anthropogenic contribution for copper, zinc, arsenic, silver, cadmium, and lead and with arsenic identified as the principal long-term health concern (85). Polycyclic aromatic hydrocarbons of combustion origin have been documented in the same particulate fractions (67).

The crustal-versus-combustion problem, stated properly: Desert dust is largely crustal in origin—silicates, carbonates, and lithogenic metals—and its toxicity profile differs from that of combustion-derived urban particulate, which carries elemental and organic carbon, PAHs, and anthropogenically enriched transition metals. This is not a footnote to the regional argument; it determines what the regional study can and cannot test, and it, therefore, belongs at the front of the design rather than at the end of the discussion.

Three implications follow. First, total PM_10_ in this setting is a poor exposure variable for the inhalational hypothesis as formulated in Section 8 because much of its mass is crustal and the mechanistic relevance of that fraction differs from combustion-derived particulate. A study relating MS only to total PM_10_ mass could, therefore, miss a source-specific effect. Second, the combustion-related fraction is present and separable and is the fraction most directly implicated by the AhR hypothesis: PM_2.5_ in Riyadh is substantially lower than PM_10_ and relatively more influenced by combustion-related constituents, PAHs are documented in both fractions (67), and enrichment analysis identifies an anthropogenic contribution for copper, zinc, arsenic, silver, cadmium, and lead (85), distinguishing them from the lithogenic background of iron, chromium, and cobalt. Third, the divergence between crustal and combustion sources is large enough to be used as an internal contrast rather than treated simply as noise. Source apportionment—for example, positive matrix factorization applied to speciated data or enrichment factors and NO_2_ as combustion tracers against silicon or aluminum as crustal tracers—could assign residential exposure separately for crustal and combustion factors within the same population. In many European urban datasets, these source contributions can be more tightly correlated; the Gulf’s large crustal contribution may provide a wider internal source contrast. The prediction is explicit: an association with the combustion factor but not the crustal factor would support the AhR-mediated mechanism, whereas an association with total mass alone, or with the crustal factor, would point to a different mechanism such as generic innate immune activation and would count against claim 2.

The convergence of metals is similarly specific rather than generic. Arsenic and cadmium are precisely the two metals for which the largest biomonitoring meta-analysis found the most consistent elevation in MS (49), and in Riyadh they arrive as anthropogenically enriched constituents of a particulate burden that also carries combustion products, in a population with substantial petrochemical employment. The chief methodological obstacle in the metals literature—that metals cannot be isolated from particulate and industrial co-exposure—is here not an obstacle but the object of study. This is what a mixture, in the sense the exposome literature intends (51, 76), looks like.

### 10.4 Disease occurrence and existing assets

The outcome is moving, though the evidence is largely for prevalence, with clear longitudinal incidence data available for particular countries rather than the region as a whole. MS was historically classified as low risk across the Arabian Gulf under the Kurtzke schema; regional consensus recognized more than a decade ago that this no longer held (88). The Saudi national registry projected prevalence of 40.40 per 100,000 total population and 61.95 per 100,000 among Saudi nationals (82), Kuwait’s national registry has documented continued increase, and its interrupted time-series analysis provides the region’s clearest incidence trend (79, 83), and Qatar and the UAE report comparable or higher prevalence (89). Part of this rise reflects ascertainment, as the Kuwaiti work makes explicit (79). However, ascertainment artifact and true incidence change are separable with registry data, and that separation has not been seriously attempted region-wide.

Regional environmental MS research is not absent. A matched multicenter case–control study of 307 Saudi patients and 307 controls across three cities examined sun exposure, tobacco, diet, consanguinity, and coffee, reporting protective associations for high sun exposure during primary school and university and for fruit and coffee consumption, and increased risk with fast-food intake (75); a Kuwaiti case–control study examined sociocultural and demographic factors (90). Both are questionnaire-based. Neither incorporated measured ambient exposure, particulate speciation, occupational exposure reconstruction, or genotyping. That is the gap.

What exist, then, are as follows: national MS registries in Saudi Arabia and Kuwait (79, 82, 83), long-term multi-site ambient monitoring data for Riyadh (84), speciated particulate composition data including metals and PAHs (67, 85), HLA genotyping capacity in regional tertiary centers, and established multicenter recruitment networks demonstrated by the existing case–control work (75, 90). What does not exist is any linkage between them, and there is no pre-onset biobank. **Table 2** sets out the resulting agenda by exposure domain, including the design specification developed below.

**Table 2:** A regional research agenda: the Gulf exposome.

Exposure domain	Regional characteristics	Existing assets	Unresolved question	Feasible design, with specification
Ambient particulate matter	Annual PM_2.5_, PM_10_, NO_2_ above WHO guidelines every year 2015–2023; PM_10_ dominated by desert dust; divergent pollutant trends (84)	Nine years of data across 13 fixed monitoring stations, Riyadh (84)	Does the NO*_x_* dose–response reported in Sweden (31) extend to an exposure range far above the sampled ceiling?	Registry-based case–control, incidence-density sampled; population controls from the civil registry matched on birth year, sex and nationality stratum; dispersion-modeled residential exposure over ages 5–19 (primary window) with 10-year and lifetime windows pre-specified as secondary; HLA stratification
Metals within particulate	PM_10_ 223 µg/m³ vs. PM_2.5_ 35 µg/m³; Fe, Ni, Cr, Zn, Co, Cu, Ag, As, Cd, Pb quantified; anthropogenic enrichment for Cu, Zn, As, Ag, Cd, Pb; As the principal health concern (85)	Speciated PM composition data	Are As and Cd — the metals most consistently elevated in MS (49) — independent determinants or markers of particulate burden?	Source-apportioned exposure modeling (combustion vs. crustal factors) applied to the same case–control frame; pre-onset biobanked serum for prospective extension; matrix chosen to index the childhood–adolescence window (Section 6.1)
Combustion products	Petrochemical industry; polycyclic aromatic hydrocarbons documented in Riyadh PM_2.5_ and PM_10_ (67)	Regional occupational registries; established multicenter recruitment networks (75, 90)	Does the solvent/dust HLA interaction (43, 44) replicate in a different genetic background?	Job-exposure matrix applied to full occupational history of registry cases and matched controls, with genotyping; interaction pre-specified on both additive and multiplicative scales
Disease occurrence	Prevalence 40.40/100,000 (61.95 among Saudi nationals) (82); continued rise in Kuwait (83); comparable in Qatar and UAE (89)	National MS registries (Saudi Arabia, Kuwait)	How much of the observed rise is true incidence versus ascertainment (79)?	Interrupted time-series with explicit ascertainment modeling and capture–recapture; date of first clinical event recorded separately from date of diagnosis
Established confounders	Vitamin D deficiency despite high ambient UV; sun exposure protective in regional data (75); rising obesity; regional smoking and waterpipe patterns	Registry covariate fields; existing regional case–control cohorts	Can inhalational effects be separated from correlated established determinants and from differential access to care?	Pre-specified adjustment set; negative-control exposures and outcomes; distance to nearest MS center modeled explicitly rather than adjusted away; MR where instruments allow
Genetic background	HLA-DRB1*15 associated with MS in MENA but more weakly than in northern Europe; DRB1*03/*04 risk and DRB1*07/*11 protective signals reported (86); distinct national allele distribution (87)	HLA genotyping capacity in tertiary centers; national donor-registry allele frequency data (87)	Is the reported HLA-restricted interaction a property of the allele and exposure, or of the Swedish source population?	Genotyping nested in the case–control study, powered for interaction with DRB1*15 and with regionally frequent alleles
Mechanism	Exposure routes of Section 8 are all measurable in this setting	Tertiary immunology laboratories; MRI capacity	Do the predicted immunological gradients accompany the exposure gradient?	Nested exposome–immunophenotype substudy as specified in **Table 3**

The table shows what regional data already exist and what a feasible design would require, per exposure domain. It complements rather than duplicates **Table 1**: where **Table 1** grades global evidence, this table identifies where the Gulf setting can contribute new evidence. Cells describe assets or design features rather than reporting effect estimates.

WHO, World Health Organization; MR, Mendelian randomization; UV, ultraviolet; PM, particulate matter; HLA, human leukocyte antigen; MENA, Middle East and North Africa.

### 10.5 What kind of study: natural experiment or observational comparison?

The phrase “natural experiment” is used loosely in this literature, and the distinction matters for what can be concluded.

A natural experiment in the strict sense requires an exogenous change in exposure—a policy, plant closure, fuel standard, or regulatory intervention—that is plausibly unrelated to the determinants of the outcome and can be exploited by interrupted time-series, difference-in-differences, or regression-discontinuity designs. Documented or future policy changes in the Gulf may create such opportunities, and the Kuwaiti interrupted time-series demonstrates that regional registry data can support quasi-experimental analysis (79). Secular pollutant trends alone, however, are not exogenous interventions and should not be labeled natural experiments. Diagnostic practice and other contemporaneous changes would remain central threats to causal interpretation.

What the region principally offers is not a natural experiment but a *natural contrast*: a wide, measured, and partly source-decomposable exposure gradient in a population with different genetic background and different correlated exposures from those in which the hypothesis was generated. That is an observational design, and it should be described as such.

Accordingly, the primary proposal is a registry-based case–control study with incidence-density sampling. Its specification, rather than a generic label, is what determines whether it is informative.

Cases: Incident MS cases from the national registry meeting current diagnostic criteria, with date of first clinical event recorded separately from date of diagnosis, since the two differ systematically as diagnostic access improves.

Controls: Population controls sampled at random from the national civil registry, individually matched to cases on year of birth, sex, and nationality stratum, and sampled by incidence density so that each control is at risk at the index case’s event date. Hospital controls should be avoided: care-seeking in tertiary centers is itself associated with urban residence, and urban residence is the principal confounder of interest. Where a national sampling frame cannot be accessed, a second-best is neighborhood-matched controls with residential history collected identically to cases—but neighborhood matching removes the between-district exposure contrast that the study exists to exploit and would have to be restricted to matching on socioeconomic indicators rather than location.

Exposure window: Residential and occupational history should be reconstructed for the whole period from age 5 to the index date, with exposure summarized in three pre-specified windows: childhood and adolescence (ages 5–19), the 10 years preceding the index date, and lifetime cumulative exposure. The primary analysis should be pre-specified as one window; on current evidence for established determinants (Section 8.6), the childhood-and-adolescence window is the defensible primary choice for susceptibility, with the 10-year window as the primary choice for any analysis of relapse or MRI activity. Reporting all three without pre-specification would invite selective emphasis.

Exposure assignment: Dispersion-modeled or land-use-regression concentrations assigned to each residential address, with separate source-apportioned factors for combustion and crustal contributions (Section 10.3), plus a job-exposure matrix applied to occupational history for solvents, dust, and metals. Ambient assignment should be accompanied by a personal-monitoring validation substudy in a sample of participants (Section 9) since the ratio of personal dose to modeled ambient concentration is unknown in this setting and may differ markedly from European estimates.

Confounding: Pre-specified adjustment for smoking (including waterpipe use, which is regionally prevalent and poorly captured by cigarette-based instruments), adolescent body mass index, vitamin D status, EBV serostatus where available, education and household income, nationality stratum, and district-level socioeconomic indicators. Where credible candidates can be specified, negative-control exposures and outcomes should be pre-specified to probe residual confounding; distance to the nearest MS center should be modeled explicitly because differential diagnostic access is part of the causal structure rather than a generic nuisance covariate.

Interaction: HLA genotyping of cases and controls, with pre-specified analysis of interaction with DRB1*15 (and, given the regional allele distribution, with DRB1*03 and DRB1*04) on both additive and multiplicative scales, reported with the scale stated.

### 10.6 From exposure to mechanism to measurable endpoint

A regional proposal that stops at an odds ratio would reproduce the limitation identified throughout this review: an association without a mechanism. The design above should, therefore, be paired with a nested exposome–immunophenotype substudy in which the molecular routes of Section 8 are measured directly. **Table 3** sets out, for each route, the regional exposure, the molecular node, the measurable human endpoint, the directional prediction, and the observation that would count against it. Three features of that table are worth stating explicitly.

**Table 3:** Testable mechanistic predictions for a Gulf exposome study.

Molecular route (Section 8)	Regional exposure to be measured	Human endpoint	Directional prediction	Observation that would count against the model
AhR ligation (61–66)	Combustion-apportioned PM_2.5_ and particulate PAH burden (67, 85); urinary PAH metabolites as internal dose	CYP1A1/CYP1B1 induction in peripheral blood mononuclear cells; Th17 frequency and IL-17A production; Th17:Treg ratio	Graded increase in AhR target-gene induction and Th17:Treg ratio across the combustion-exposure gradient, stronger in DRB1*15 carriers	No gradient in target-gene induction despite a demonstrated PAH gradient; or a gradient tracking crustal rather than combustion apportionment
SCFA availability (55–58, 68–70)	Dietary fiber intake; adolescent and current BMI	Serum and fecal acetate, propionate, butyrate; Foxp3^+^ Treg frequency and suppressive function	Lower SCFA concentrations in cases than in matched controls, with the difference tracking fiber intake rather than disease duration or therapy	SCFA differences explained by disease duration, disability, or disease-modifying therapy rather than by diet
Oxidative and metabolic stress (47, 70)	Speciated As and Cd exposure; matrix chosen for the relevant window (Section 6.1)	Treg mitochondrial respiration; systemic oxidative-stress markers	Impaired Treg suppressive function and mitochondrial respiration in the highest exposure stratum	Impairment present but unrelated to measured metal exposure, or attributable to disease activity
EBV-associated mimicry (60, 72)	EBV serostatus and anti-EBNA1 titer in cases and controls	Anti-EBNA1 and anti-GlialCAM antibody reactivity	Environmental exposure modifies the association between anti-EBNA1 reactivity and disease, rather than acting independently of it	Exposure and EBV reactivity act additively with no evidence of modification
Lung–CNS axis and barrier disruption (41, 73)	Combustion-apportioned exposure; occupational inhalational exposure	Serum neurofilament light chain and GFAP; new T2 and gadolinium-enhancing lesions	Exposure gradient accompanied by a gradient in subclinical neuroaxonal injury markers and MRI activity	No relationship between exposure and neuroaxonal injury markers in the presence of a clinical association

Endpoints are ordered within each row by feasibility with existing regional laboratory capacity. No result in this table has been observed; the table states predictions and their negative cases.

AhR, aryl hydrocarbon receptor; BMI, body mass index; EBV, Epstein–Barr virus; GFAP, glial fibrillary acidic protein; PAH, polycyclic aromatic hydrocarbon; SCFA, short-chain fatty acid; Treg, regulatory T cell.

First, each prediction names an immunological or neurological quantity that can be measured in blood, stool, or on MRI, rather than a general claim about inflammation. Second, each route has a specified negative case so that the model can fail. Third, the predictions are ordered by feasibility: AhR target-gene induction and SCFA quantification require only peripheral blood and stool with standard assays, whereas Treg mitochondrial respirometry and untargeted exposomics require dedicated capacity.

The mechanistic substudy also disciplines the epidemiology. If the combustion factor is associated with MS but is not associated with CYP1A1/CYP1B1 induction or with a Th17 shift in the same population, then either the mechanism is wrong or the exposure assignment is too crude, and the two possibilities are distinguishable. If the crustal factor is associated with MS while the combustion factor is not, the AhR account is not supported and an alternative—innate immune activation by mineral particulate, for which there is a separate literature—becomes the leading candidate. Neither outcome is a failure of the study.

### 10.7 What would falsify the working model

To be usable, the model of Section 1.2 must be able to fail. It would be substantially weakened by any of the following: absence of a dose–response for the combustion-apportioned exposure across a range far wider than the Swedish one, after adjustment for the confounders specified above; absence of HLA-restricted interaction in a population with adequate power and a different allele distribution; a pattern in which total or crustal particulate mass, but not the combustion fraction, carries the association; or absence of the predicted immunological gradients in **Table 3** despite a demonstrated exposure gradient. Each of these is an achievable observation, not a rhetorical concession.

Finally, there are three caveats that a regional study must address explicitly rather than acknowledge in passing. Regional vitamin D deficiency despite high ambient ultraviolet exposure, rising obesity, and regional smoking patterns are substantial confounders—and the Saudi case–control finding that sun exposure is protective (75) indicates that these determinants are operative regionally rather than merely imported assumptions. Rapid change in diagnostic access means any incidence-based analysis must model ascertainment rather than assume it away (79), and the crustal fraction that dominates PM_10_ mass means that source apportionment is not an optional refinement of this design but a precondition for it (Section 10.3).

## 11 Methodological challenges and causal inference

Several limitations recur across this literature and explain much of its inconsistency.

Reverse causation dominates cross-sectional biomonitoring, where measured exposure may be a consequence of established disease. This applies with equal force to metals (48, 49), persistent organic pollutants (51), and the microbiome (52, 54), and it is why those three domains sit below the inhalational domain in **Table 1** despite comparable or larger point estimates.

Exposure misclassification takes a different form in each domain: residential proximity is a crude proxy for personal dose (45, 46); biological matrices index different temporal windows, as Section 6.1 sets out in detail; and binary ever/never occupational self-report cannot support dose–response (43, 44, 47).

Residual confounding is difficult to eliminate because exposures cluster. Smoking is critical for all inhaled exposures and must be adjusted explicitly (9, 31). Socioeconomic status, occupation, healthcare access, and urban residence influence both exposure and ascertainment. Diagnostic evolution compounds the problem for any historical analysis: revised criteria, MRI availability, growing neurological expertise, and registry development generate apparent secular increases, as the Kuwaiti analysis demonstrates (79).

Non-independence of evidence is a further problem, and one this review has flagged repeatedly rather than once. A substantial share of the gene–environment interaction literature in MS derives from a small number of Swedish population-based datasets analyzed by overlapping investigator groups (31, 43, 44, 51). Consistency among such studies is weaker evidence than consistency among independent populations and should not be interpreted as independent replication.

Publication bias is an under-appreciated distortion: positive findings are more likely to be published than null results, and pre-registration remains uncommon in observational environmental epidemiology (30–33). The discordance between positive Swedish NO*_x_* findings and null US cohort studies may partly reflect this asymmetry. We caution against treating the published record as an unbiased sample of conducted studies and note that the regional proposal in Section 10 would be strengthened by pre-registration of exposure windows, outcomes, and interaction models to protect against selective reporting.

Against these, the field has under-used the designs that address them and used one of them incompletely. Mendelian randomization has transformed the vitamin D and obesity literatures (12, 15–17) and has been applied broadly to candidate MS risk factors (23–25), but it has scarcely been applied to pollutant exposures, where instruments are admittedly harder to construct. The smoking case (Section 3) is instructive in both directions: MR can challenge an observational consensus, but a null MR result with weak instruments is not equivalent to a demonstrated absence of effect, and MR of main effects is poorly suited to exposures acting chiefly within a genotype stratum. Triangulation across designs with non-overlapping biases, negative-control exposures and outcomes, and target trial emulation using natural experiments in air-quality policy are all available and largely untried in MS. The discrepancy between prospective and case–control studies in the pollution literature (Section 4) is precisely the kind of question triangulation exists to resolve.

Finally, traditional studies evaluate one exposure at a time despite real-world co-exposure. The PFAS mixture analysis (51) shows what is missed: combined exposure raised risk where individual compounds did not. This is the strongest methodological argument for exposome-based approaches, and it is empirical rather than rhetorical (28, 76).

## 12 Clinical and public health implications and future directions

Smoking cessation remains the clearest actionable intervention among established modifiable exposures (8, 9). The null MR findings (19, 20) do not alter this recommendation, since smoking cessation is justified many times over on other grounds and the observational MS evidence, including gene–environment interaction, remains substantial. Prevention of adolescent obesity is strongly justified. Low vitamin D status is supported as a causal susceptibility factor by Mendelian randomization (12, 16), but this should not be interpreted as proof that vitamin D supplementation prevents MS; correction of deficiency remains appropriate on general clinical grounds. Recommendations for the emerging exposome should remain proportionate to evidence quality: no current data justify chelation, detoxification regimens, or individual avoidance recommendations based on unvalidated biomonitoring signals.

Priorities for research follow directly from the limitations above. First, prospective cohorts with pre-onset exposure assessment and biobanked serum, which will enable the metals, PFAS, and microbiome questions to be asked with correct temporality (28, 51, 54). Second, exposure characterization at the individual level—this includes satellite-derived estimates, dispersion modeling, geographic information systems, personal monitoring, and job-exposure matrices integrated with residential and occupational histories (31, 76, 84). Third, causal designs: Examples include Mendelian randomization where instruments permit, triangulation across designs, negative controls, and target trial emulation of air-quality interventions (23). Fourth, mixture-aware analysis should be considered as the default rather than the exception (51). Fifth, it is worth conducting replication in populations outside the northern European settings, where most positive findings originate—which returns to Section 10.

Ultimately, the clinically meaningful question is not whether an exposure is associated with MS, but whether reducing it changes risk or inflammatory activity. No study reviewed here answers that question. Designing one that can is the field’s central task.

## 13 Conclusions

The environmental contribution to MS extends well beyond its established determinants, but evidence strength varies by more than an order of magnitude across exposures, and reviews that present them as a single category obscure this.

Inhalational exposures carry the most coherent signal. Long-term particulate matter shows modest but consistent pooled associations (PM_2.5_ HR 1.21, 95% CI 1.07–1.38) (30), combustion-related NO*_x_* shows dose-dependence and HLA synergy (31), and organic solvents (43) and industrial dust (44) show genotype-restricted interaction patterns similar to that established for smoking. The recurrence of broadly similar genotype-restricted interaction patterns across chemically distinct inhaled agents is among the more compelling observations in this literature, though it is qualified by three things that should be reported rather than omitted: the two largest prospective cohorts were null (32, 33), Mendelian randomization does not support a causal effect of smoking on current instruments (19, 20), and the interaction findings derive from related Swedish datasets and have not been independently replicated elsewhere (31, 43, 44, 51).

Heavy metals remain biologically plausible but rest predominantly on cross-sectional data vulnerable to reverse causation, and on matrices that index exposure windows other than the etiologically relevant one (47–50). Persistent organic pollutants are an emerging signal whose principal contribution so far is methodological: mixture effects appear where single-compound effects do not (51). Residential proximity to industry shows inconsistent associations limited by exposure proxy quality (45, 46). Diet and the microbiome remain promising and are now supported by transferable, metabolite-mediated experimental effects but remain temporally unresolved in humans (52, 54–58). Contemporary evidence does not support the popular narrative that the 1990–1991 Gulf War increased MS risk (77–79).

Environmental exposures rarely act independently. Their effects converge toward a limited set of molecular nodes—AhR ligation, SCFA availability, EBV-associated mimicry, and redox-dependent Treg function—operating on very different time scales and conditioned by HLA-restricted susceptibility (3). These observations support the MS exposome as a framework. But a framework earns its place by generating testable predictions in settings that can discriminate between hypotheses.

The Gulf region may represent such a setting: rising prevalence and, where measured, incidence; an exposure ceiling far above the range in which the dose–response was characterized; a particulate burden in which the implicated combustion fraction is separable from a dominant crustal one; arsenic and cadmium co-delivered with combustion products in a single inhaled matrix; an HLA distribution different from the northern European populations in which the interaction findings were generated; and national registries and monitoring networks already in place, with regional environmental research so far confined to questionnaires (75, 82, 84–87). Section 10 specifies the design, the endpoints, and the results that would count against the model. Making that link, and stating in advance what would refute it, is the most tractable way to move the MS exposome from framework to measurement.
